# Interaction of Psoriasis and Bullous Diseases

**DOI:** 10.3389/fmed.2018.00222

**Published:** 2018-08-08

**Authors:** Teruki Dainichi, Kenji Kabashima

**Affiliations:** ^1^Department of Dermatology, Kyoto University Graduate School of Medicine, Kyoto, Japan; ^2^Singapore Immunology Network and Institute of Medical Biology, Agency for Science, Technology and Research (A^*^STAR), Singapore, Singapore

**Keywords:** autoimmunity, Th2, Th17, psoriasis, pemphigoid, laminin, MMP, senescence

## Abstract

Patients with psoriasis are frequently complicated with autoimmune bullous diseases, especially, pemphigoid diseases. It has been known that one-third cases of anti-laminin gamma1 pemphigoid, formerly anti-p200 pemphigoid, are associated with psoriasis whereas bullous pemphigoid is the most frequently associated bullous disease in psoriasis cases regardless of the lack of detectable levels of the accompanying anti-laminin gamma1 autoantibodies. Despite several suggestions, however, the definitive reason of the striking association of psoriasis and these autoimmune bullous diseases remains elusive. In this review, we look over the epidemiological evidence of the association of psoriasis and autoimmune bullous diseases and the information of genetic susceptibilities of each disease, and discuss the possible mechanisms of their complication with reference to the recent understandings of each pathogenesis.

## Introduction

Autoimmune bullous diseases, as well as psoriasis, are skin disorders affecting the epidermis. In both diseases, immune reactions target the epidermis, and induce the development of the skin lesions following the failures in epithelial cell contacts or the defects in epithelial cell proliferation and differentiation. There is remarkable progress in the understandings of their pathogenesis in these decades, respectively. Nevertheless, (1) what triggers the pathogenic immune reactions, (2) which cells by which molecules respond to the internal or external changes and direct the subsequent immune reactions, and (3) which step is critical for the decision of the immune type, have not yet been fully elucidated.

Physicians and dermatologists have long time been aware that psoriasis patients are frequently complicated with autoimmune bullous diseases. Indeed, epidemiological evidence indicates that the incidence of some pemphigoid diseases in psoriasis patients is significantly higher than that in the control individuals without psoriasis. Moreover, recent investigations have suggested that there are in part similarities and shared players in their pathogenesis.

In this review, first we look over the epidemiological evidence of the association of psoriasis and autoimmune bullous diseases. Second, we compare their genetic susceptibilities. And third, we discuss the possible mechanisms of their association with reference to the current understandings on each pathogenesis.

## Epidemiological evidence

### Psoriasis and pemphigus

Most reported cases of pemphigus developed in psoriasis patients were pemphigus foliaceus including pemphigus erythematosus. A case series of 145 patients with concomitant psoriasis and autoimmune blistering diseases from Japan reported that all four (2.8%) pemphigus cases with psoriasis were pemphigus foliaceus (124). The first case-control study of 51,800 psoriasis patients from Taiwan demonstrated the significantly higher prevalence rate of pemphigus in the patients than that in the control subjects (odds ratio (OR), 41.8; 95% confidence interval (CI), 12.4–140.9; *P* < 0.0001) (125).

There is another study evaluating their association in an inverse direction: a case-control study of 1985 pemphigus patients from Israel demonstrated that the prevalence rate of psoriasis in pemphigus patients was also higher than that in the controls (OR, 2.84; 95% CI, 2.09–3.85, *P* < 0.001) (126).

### Psoriasis and pemphigoid diseases including epidermolysis bullosa acquisita

**Figure 1:**
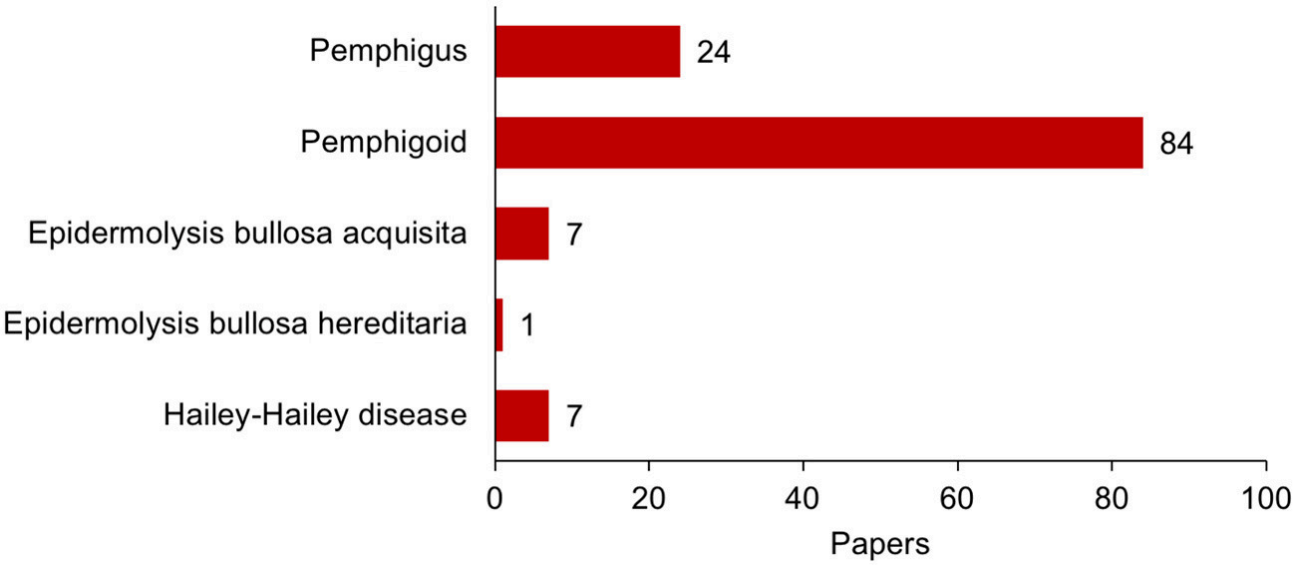
Publication of the report of cases with association of psoriasis and bullous diseases until the end of 2017. All the publications were searched in PubMed database, and case reports and case series were selected manually with exclusion of redundancy. Cases with coexistence of two or more autoimmune blistering diseases were counted in each category: (psoriasis[tiab] AND pemphigoid[tiab]) for pemphigoid diseases; (psoriasis[tiab] AND pemphigus[tiab] NOT pemphigoid[tiab]) for pemphigus; psoriasis[tiab] AND (epidermolysis bullosa acquisita[tiab]) for epidermolysis bullosa acquisita; psoriasis AND (epidermolysis bullosa hereditaria OR epidermolysis bullosa simplex OR junctional epidermolysis bullosa OR dystrophic epidermolysis bullosa OR kindler's syndrome OR kindler syndrome) for epidermolysis bullosa hereditaria; psoriasis[tiab] AND (hailey-hailey OR familial pemphigus OR familial benign chronic pemphigus) for Hailey-Hailey disease. References are as follows. Pemphigus (24): 1990 or earlier (5) (1–5); 1991–2000 (5) (6–10); 2001–2010 (6) (11–16); 2011 or later (8) (17–25). Pemphigoid (84): 1980 or earlier (7) (26–32); 1981–1990 (16) (3, 33–47); 1991–2000 (10) (48–57); 2001–2010 (21) (58–78); 2011 or later (30) (79–109). Epidermolysis bullosa acquisita (7) (51, 110–115). Epidermolysis bullosa hereditaria (1) (116). Hailey-Hailey disease (7) (117–123).

**Figure 2:**
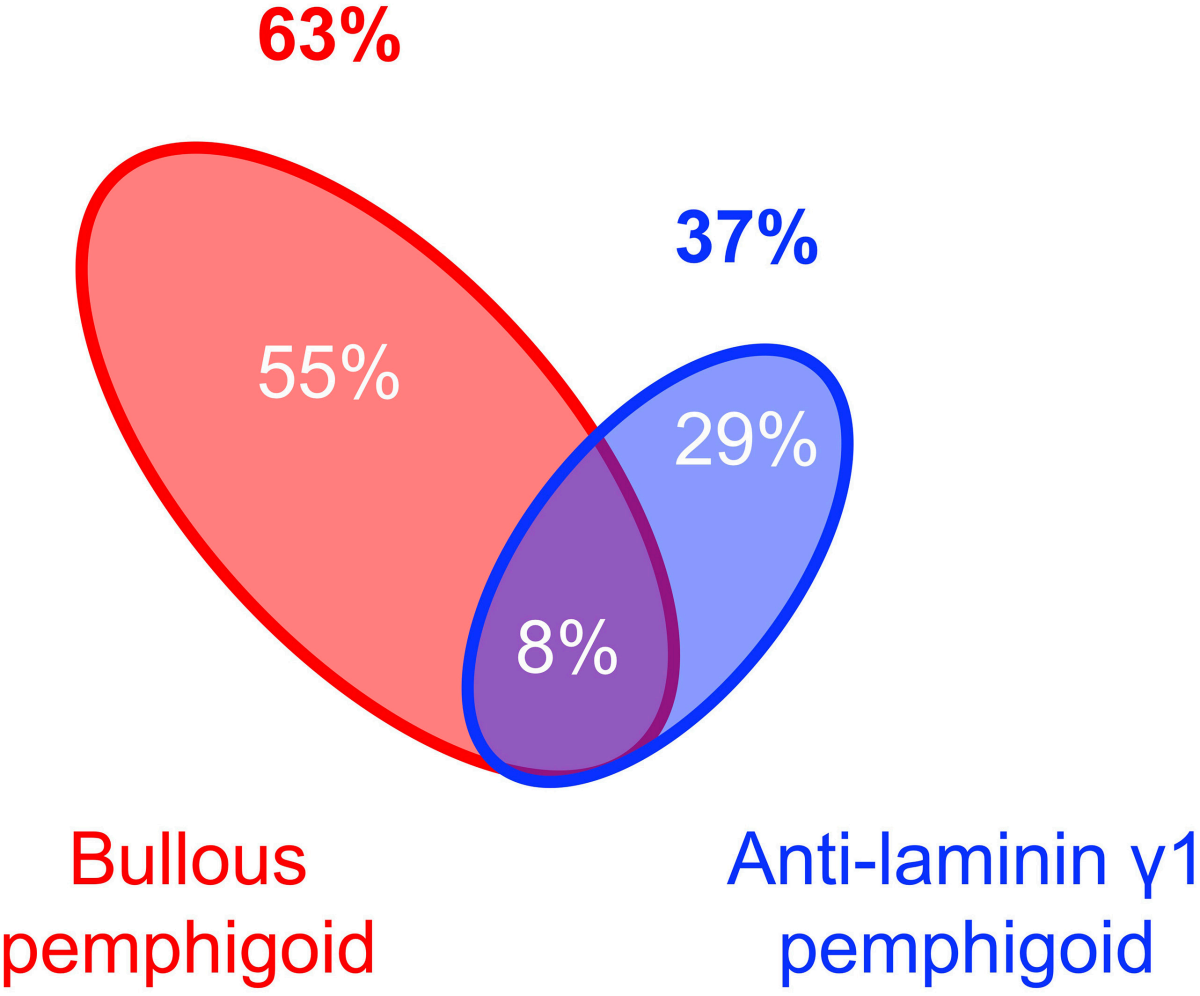
Component of pemphigoid diseases associated with psoriasis (124).

Complication of psoriasis cases with pemphigoid diseases are much more commonly experienced than those with pemphigus whereas the number of the report of the psoriasis cases with pemphigoid is only about three times as many as those with pemphigus (Figure 1). Indeed, in the case series of 145 patients with psoriasis and autoimmune blistering diseases from Japan, almost all the cases are complicated with bullous pemphigoid (63%), anti-laminin γ1 pemphigoid (formerly anti-p200 pemphigoid) (37%), or their combination (8%) (Figure 2). Psoriasis including pustular psoriasis precedes the development of pemphigoid in most cases. Of note, 111 (78.7%) cases had no history of any phototherapies in this case series (124).

The case-control study of 51,800 psoriasis patients from Taiwan also demonstrated the higher prevalence rate of pemphigoid in the patients than that in the control subjects (OR, 14.8; 95% CI, 5.00–43.50, *P* < 0.0001) (125).

Inversely, early case-controlled study has shown that 7 out of 62 (11%) pemphigoid cases are complicated with psoriasis and the prevalence was significantly higher than expected in the controls (*P* < 0.01) (40). Following studies also confirmed that psoriasis cases are significantly associated with bullous pemphigoid: A study of 3,485 bullous pemphigoid cases from Taiwan (OR 2.02; 95% CI 1.54–2.66, *P* < 0.003) (127), and another of 287 bullous pemphigoid cases from Israel (OR 4.39; 95% CI 2.17–8.92, *P* < 0.0001) (128), respectively.

Anti-laminin γ1 pemphigoid is originally reported as pemphigoid developed in psoriasis patients with circulating autoantibodies against unknown autoantigen. Around one-third of the following cases have also been associated with psoriasis (129).

There are only a few reported cases of psoriasis associated with other pemphigoid diseases. The case series of 145 patients with psoriasis and autoimmune blistering diseases from Japan included three cases with linear IgA bullous dermatosis and two cases with epidermolysis bullosa acquisita (124). There are few independent reports of a case with epidermolysis bullosa acquisita (51, 112), or with anti-laminin 332 mucous membrane pemphigoid (109).

### Psoriasis and other blistering diseases

Intriguingly, as far as we looked up, there is no reported case of psoriasis in any type of epidermolysis bullosa: simplex, junctional, or dystrophic type, except for one case report of the dystrophic type without confirmation by DNA sequencing analysis (116). There are seven reports of a case with psoriasis in Hailey-Hailey disease since the first reported case (117).

## Susceptibilities of psoriasis and bullous diseases

### HLA

No shared susceptibility human leukocyte antigen (HLA) alleles have been reported between psoriasis and bullous diseases that can be associated with psoriasis: HLA-Cw^*^0602 allele has been identified in psoriasis susceptibility 1 (*PSORS1*), a major psoriasis susceptibility locus (130). On the other hand, HLA-DRB1 alleles, such as DRB1*1401, DRB1*0402, and DRB1*08 alleles are associated with pemphigus vulgaris (131). HLA-DQB1^*^0301 allele has been identified as a susceptibility gene for bullous pemphigoid. Epidermolysis bullosa acquisita is associated with DRB1^*^15:03 allele (132).

### Other susceptibility genes

Studies for single nucleotide polymorphisms have been defined several psoriasis susceptibility genes (130, 133) (Table 1) whereas it has been challenging to identify the susceptibility genes of pemphigus or pemphigoid diseases and there is much less information about their susceptibility genes. As for two major bullous diseases that can be associated with psoriasis, following genes are suggested to be associated with the disease susceptibility:, *IL1B* (135), *CD16* (136), *ATP8* (137), and *CYP2D6* (138) in bullous pemphigoid; and *CD40L, CD40, BLYS* (139), *CTLA4* (140), and *CD59* (141) in pemphigus foliaceus. However, they are not included in the major psoriasis susceptibility genes except for the risk loci at *IL1B* in late onset psoriasis (142). Susceptible SNPs in mucous membrane pemphigoid were recently reported (143) whereas mucous membrane pemphigoid rarely accompanied with psoriasis.

**Table 1:** SNPs in psoriasis and the related bullous diseases (130, 133).

**Disease**	**Category**	**Symbols**
Psoriasis	HLA	HLA-C*12:03, HLA-B, HLA-A, HLA-DQA1
	MHC class-I processing	*ERAP1*
	NF-κB signaling	*REL, TNIP1, NFKBIA, CARD14*
	IFN signaling	*IL28RA, TYK2*
	T-cell regulation	*RUNX3, IL13, TAGAP, ETS1, MBD2, PTPN22*
	Antiviral signaling	*IFIH1, DDX58, RNF114*
	IL-23/IL-17 axis	*TNFAIP3, IL23R, IL12B, TRAF3IP2, IL23A, STAT3*
	Th2	*IL4, IL13*
	Late cornified envelope	*LCE3B, LCE3C, LCE3D*
	Ubiquitin pathway	*ZNF313*
	Unknown	*CDKAL1*
Bullous pemphigoid		*IL1B, CD16, ATP8, CYP2D6*
multicolumn1lPemphigus foliaceus		*CD40L, CD40, BLYS, CTLA4, CD59*

### Transcriptomic studies

Whereas transcriptomic analyses are preferentially demonstrated to investigate the pathogenesis of psoriasis, it is not in the case of autoimmune bullous diseases. The increased expression levels of *CD1D* (4.0) and *LILRB2* (4.7) were reported in pemphigus foliaceus (144), neither of them were included in the upregulated genes in psoriasis lesions (134) (Table 2).

**Table 2:** Top 25 upregulated genes in the psoriasis lesions relative to the non-lesional skin (134).

**#**	**Symbol**	**Description**	**Fold change**
1	*SERPINB4*	serpin peptidase inhibitor, clade B (ovalbumin), member 4	661
2	*S100A12*	S100 calcium binding protein A12	328
3	*TCN1*	transcobalamin I (vitamin B12 binding protein, R binder family)	309
4	*S100A7A*	S100 calcium binding protein A7A	260
5	*SPRR2C*	small proline-rich protein 2C (pseudogene)	167
6	*DEFB4A*	defensin, beta 4A	138
7	*AKR1B10*	aldo-keto reductase family 1, member B10 (aldose reductase)	89
8	*PI3*	peptidase inhibitor 3, skin-derived	80
9	*IL8*	interleukin 8	66
10	*TMPRSS11D*	transmembrane protease, serine 11D	63
11	*SERPINB3*	serpin peptidase inhibitor, clade B (ovalbumin), member 3	62
12	*S100A9*	S100 calcium binding protein A9	60
13	*OASL*	29-59-oligoadenylate synthetase-like	56
14	*ATP12A*	ATPase, H+/K+ transporting, nongastric, alpha polypeptide	54
15	*LCN2*	lipocalin 2	53
16	*RHCG*	Rh family, C glycoprotein	52
17	*IGFL1*	IGF-like family member 1	48
18	*KYNU*	kynureninase (L-kynurenine hydrolase)	48
19	*IL1F9*	interleukin 1 family, member 9	43
20	*KLK6*	kallikrein-related peptidase 6	43
21	*LTF*	lactotransferrin	36
22	*CCL20*	chemokine (C-C motif) ligand 20	35
23	*C10orf99*	chromosome 10 open reading frame 99	34
24	*HPSE*	heparanase	33
25	*ADAMDEC1*	ADAM-like, decysin 1	33

Consequently, these results suggest that the complication of psoriasis with bullous pemphigoid or pemphigus foliaceus are not attributed to the shared susceptibility. Therefore, it would be more reasonable to consider that the epigenetic events in psoriasis lesions give rise to the increased rate of the complication with autoimmune bullous diseases.

## Potential mechanisms of the association of psoriasis and pemphigoid diseases

### Local inflammation

Psoriasis plaques are the frequently affected sites for the blister formation of associated autoimmune bullous diseases, such as bullous pemphigoid (58), anti-laminin γ1 pemphigoid (49), and pemphigus foliaceus (7). It would be reasonable to consider that epigenetic changes altered by psoriasis lesion may trigger or accelerate autoreactive response to specific antigens resulting in autoantibody production, blistering formation, and further positive loop of organ-specific autoimmunity (145). Whereas detailed speculations in this context are described below, it is of not that local inflammation exacerbates cutaneous manifestations in a murine autoimmune pemphigus model (146), suggesting effective recruitment of autoantibodies into psoriasis lesions and further autoimmune loop.

### Th17

**Figure 3:**
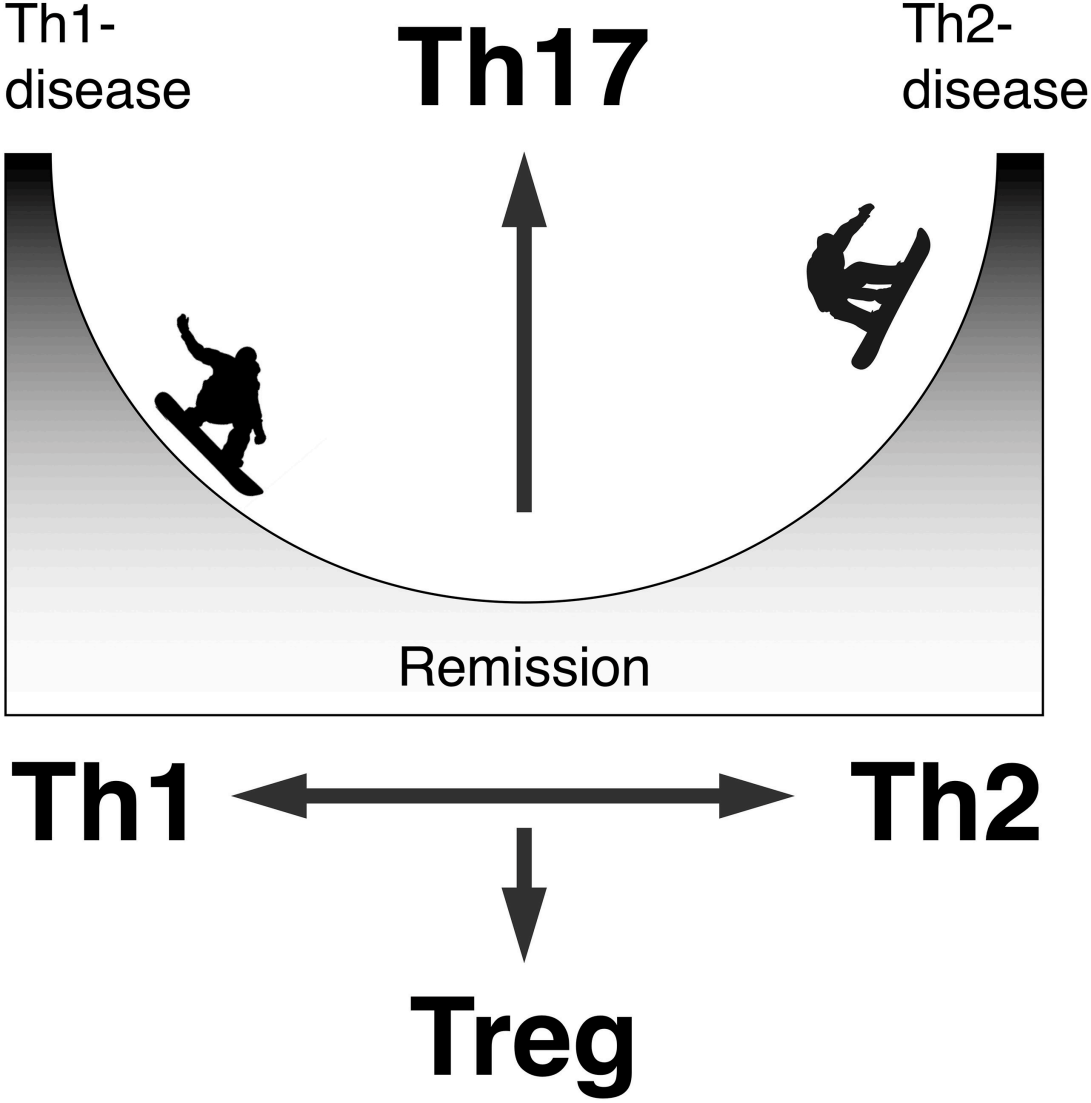
A mechanistic model to produce autoantibodies in psoriasis. Can switch from the Th1/Th17-dominant to Th2/Th17-dominant state be involved?.

There are much more psoriasis cases complicated with bullous pemphigoid than those with pemphigus. We have demonstrated that the percentages of interleukin (IL)-17+ cells in CD4+ cells in the lesional skin from bullous pemphigoid are significantly higher than those in the lesional skin from pemphigus foliaceus, and that the serum levels of IL-17 in patients with bullous pemphigoid is higher than those in healthy controls (147). Although IL-17 from T helper type 17 (Th17) cells have an essential role in pathogenesis of psoriasis, it does not explain the common order of the disease development: bullous pemphigoid following psoriasis despite the existence of a rare, inverse case: psoriasis following bullous pemphigoid (77). However, one may speculate that pathological events around the epidermis shared between psoriasis and bullous pemphigoid is related to the activation of Th17 in these diseases, and incidental switch of the immune response from Th1 to Th2 induce the production of the IgG autoantibodies resulting the complication of psoriasis with bullous pemphigoid (77) (Figure 3). Because, animal studies have demonstrated that single helper T cell clone specific for desmoglein 3 is sufficient to recapitulate autoimmune blister formation whereas the Th17-deviated T cell clone specific for desmoglein 3 induces psoriasiform dermatitis (148, 149). Occasional production of autoantibodies against BP180 and desmogleins in lichen planus cases has been reported regardless of accompanying blister formation, probably because of the consequence of interface dermatitis, suggesting Th1/Th2 dichotomy among lichen planus vs. pemphigus or pemphigoid diseases (150). In psoriasis, however, production of neither autoantibodies against BP180 nor desmogleins, but α6 integrin (151), in psoriasis has been reported without complication with blistering diseases. It is therefore unlikely that psoriasis and bullous pemphigoid or pemphigus diseases are sharing their primary effector memory T cells.

### Neutrophils and MMP

Keratinocytes in both psoriasis and bullous pemphigoid produce neutrophil chemoattractants, such as IL-8, and infiltration of neutrophil is a common histologic feature in these diseases (130, 131). Consequently, neutrophils release a series of metalloproteases, and it might be related to the substantial degradation of matrix proteins and the subsequent exposure of the antigenic epitopes from matrix autoantigens composing the dermal-epidermal junction. Specifically, a disintegrin and metalloprotease (ADAM) 9, ADAM10, and ADAM17/ tumor necrosis factor-alpha converting enzyme (TACE) degrade BP180/type XVII collagen (152), which is a major autoantigen in bullous pemphigoid while matrix metalloprotease (MMP) 2, 7, 8, 12, 14, 15, and 19 degrades laminins (153), of which trimers are targeted in anti-laminin γ1 pemphigoid (154) and anti-laminin 332 mucous membrane pemphigoid (155).

### Laminins

One may be tempted by the following idea: very high prevalence of psoriasis in anti-laminin γ1 pemphigoid can be explained by a positive loop of laminin degradation in psoriasis (129). In psoriasis, as well as in trauma or staphylococcal infections, degradation of laminin is accelerated through the increased expression levels of α5β1 integrin, fibronectin, and plasminogen activators (156). The laminin degradation is also stimulated by MMP9 released from neutrophils. Furthermore, laminin fragments stimulate the MMP9 expression. This laminin degradation loop may be contributed to decrease the threshold of spontaneous production of autoantibodies against laminin γ1 in the development of anti-laminin γ1 pemphigoid in psoriasis patients.

### Senescence

The median age of the development of bullous pemphigoid is around 80 years of age. Cell cycle and turnover of the epidermal keratinocytes are extremely accelerated in psoriasis whereas keratinocytes in psoriasis are not immortalized like carcinoma cells. Therefore, it is a plausible idea that the extracellular matrix in psoriatic skin simulates the senescent extracellular matrix and contribute to the development of bullous pemphigoid if the development of bullous pemphigoid is triggered by the senescence of the extracellular matrix produced by senescent keratinocytes. The shortened telomere lengths in psoriasis have not yet determined in keratinocytes or dermal fibroblasts, but in lymphocytes (157). In terms of senescence, type XVII collagen (BP180) changes its distribution (158) and the protein amount due to proteolysis (159) by aging. Despite several suggestions, however, the definitive reason of the predilection of bullous pemphigoid in an extremely old age remains to be elucidated.

## Concluding remarks

Epidemiological studies have confirmed that psoriasis is highly complicated by the subsequent development of autoimmune bullous diseases. The order of the disease development and the lack of shared susceptibility genes ask whether epigenetic events and molecular circumstances in psoriasis lesions raise the susceptibility to the organ-specific autoimmunity in the skin. The high prevalence of bullous pemphigoid and anti-laminin γ1 pemphigoid in patients with psoriasis promotes following investigations on the pathogenesis of each disease, especially about their unique types of immune responses, as well as the involvement of the degradation and senescence of extracellular proteins around the dermal-epidermal junctions.

## Author contributions

All authors listed have made a substantial, direct and intellectual contribution to the work, and approved it for publication.

### Conflict of interest statement

The authors declare that the research was conducted in the absence of any commercial or financial relationships that could be construed as a potential conflict of interest.
